# Two machine learning-derived nomogram for predicting the occurrence and severity of acute graft-versus-host disease: a retrospective study based on serum biomarkers

**DOI:** 10.3389/fgene.2024.1421980

**Published:** 2024-11-08

**Authors:** Qiang He, Xin Li, Yuan Fang, Fansheng Kong, Zhe Yu, Linna Xie

**Affiliations:** ^1^ Shandong Cancer Hospital and Institute, Shandong First Medical University and Shandong Academy of Medical Sciences, Jinan, Shandong, China; ^2^ Department of Hematology, The General Hospital of Jinan Military, Jinan, Shandong, China; ^3^ Department of Hematology, Affiliated Hospital of Shandong University of Traditional Chinese Medicine, Jinan, Shandong, China

**Keywords:** acute graft-versus-host disease, diagnosis, machine learning, nomogram, prediction model

## Abstract

**Background:**

Acute graft-versus-host disease (aGVHD) is a common complication after allogeneic hematopoietic cell transplantation (allo-HSCT), with high morbidity and mortality. Although glucocorticoids are the standard treatment, only half of patients achieve complete remission. Thus, there is an urgent need to screen biomarkers for the diagnosis of aGVHD to assist in the identification of individuals at risk of aGVHD. This study was to construct prediction models for the occurrence and severity of aGVHD using two machine learning algorithms based on serum biochemical data.

**Methods:**

Clinical data of 120 patients with hematological diseases who received allo-HSCT were retrospectively analyzed. Seventy-six patients developed aGVHD, including 56 grade I/II and 20 grade III/IV. First, 15 serum biochemical indicators were considered as potential risk factors, and the differences in the levels of indicators between non-aGVHD and aGVHD were observed, followed by evaluation of the diagnostic property. Subsequently, to develop the prediction models for the occurrence and severity of aGVHD, LASSO and random forest (RF) analyses were performed with experimental indicators. Finally, Venn diagram analysis was utilized to obtain shared biomarkers in the two algorithms to construct the nomogram. The model performance was measured by calibration curves. Internal and external validations were performed based on risk score models and ROC curve analyses.

**Results:**

Total 12 of 15 indicators exhibited significant differences between the aGVHD and non-aGVHD groups, with AUC values > 0.75. In machine learning analysis, eight features (LAG-3, TLR-2, PD-L1, IP-10, elafin, REG-3α, ST2, TIM3) and seven variables (LAG-3, TLR-2, PD-1, Flt_3, IL-9, elafin, TIM3) were selected to distinguish aGVHD vs. non-aGVHD as well as grade I/II vs. III/IV, respectively. Further, the corresponding nomogram models were established and calibration curves showed that prediction was in good agreement with the actual probability. Biomarker-based risk score model was constructed, which obtained AUC value >0.89 in internal and external datasets.

**Conclusion:**

Clinical variables screened through learning algorithm can predict the risk and severity of aGVHD. Our findings may help clinicians develop more personalized and reasonable management strategies.

## Introduction

Allogeneic hematopoietic stem cell transplantation (allo-HSCT) is an effective treatment option for many hematological malignancies and immune diseases (Patriarca et al., 2017). After transplantation, T-cell activation from allogeneic donors attacks not only residual malignant cells but also target organs and tissues of the host, thereby causing graft-versus-host disease (GVHD) and endangering the patients’ life (Lin et al., 2021). Statistically, acute GVHD (aGVHD, mainly involving the skin, gastrointestinal tract, and liver) occurs in about 30%–50% of patients with allo-HSCT, of which about 14% of patients may develop severe aGVHD (grade III-IV), resulting in a 1-year survival rate of only 40% in severe patients (Zeiser and Blazar, 2017). Hence, the emergence of aGVHD greatly limits the success of allo-HSCT and is a major hurdle to overcome in improving allo-HSCT safety. Despite the fact that glucocorticoids and ruxolitinib have been approved by the FDA for the aGVHD treatment, hormone resistance or unsatisfactory remission rates still occur in approximately 50% of patients (Martini et al., 2022; Algeri et al., 2023). Therefore, the search for specific biomarkers to diagnose aGVHD is a critical step in improving patient prognosis and developing novel therapeutic strategies.

GVHD is a complex process, and its occurrence and progression involve the excessive release of multiple inflammatory cytokines (Mohty and Gaugler, 2008). According to the pathology of aGVHD, potential driver markers implicate various processes, including acute-phase responses, TH1 (T helper 1) and anti-inflammatory cytokines, lymphocyte transport molecules, and other circulating markers (Gooptu and Koreth, 2020). An increasing number of studies have been devoted to exploring serum biomarkers of aGVHD. For example, genes engaged in immune cell and allogeneic responses, including CASP1 (caspase 1), CD52 (cluster of differentiation 52), FOXP3 (forkhead box P3), and ICOS (inducible T cell costimulatory), have been identified as the most valuable biomarkers of aGVHD (Cuzzola et al., 2012); plasma levels of IL (interleukin) 6, ST2 (suppression of tumorigenicity 2), TIM3 (T cell immunoglobulin and mucin domain-3), and sTNFR1 (soluble TNF receptor 1) help to predict severe GVHD and non-relapse mortality (McDonald et al., 2015); moreover, REG3α (regenerating family member 3 alpha) and ST2 (also known as MAGIC algorithm) are considered as plasma markers of gastrointestinal GVHD that predict the risk of refractory GVHD and resistance to therapy (Hartwell et al., 2017). These evidences reveal that screening of specific biomarkers from peripheral blood is a clinically valuable diagnostic tool that both avoids invasive procedures and helps guide personalized treatment after transplantation. However, the search for predictive markers in the serum of patients with aGVHD is still lacking, and the identified clinical indicators have limited diagnostic information in aGVHD.

At present, machine learning algorithms are an emerging field that can effectively prevent overfitting caused by multiple variables and explore disease-related biomarkers. It has been widely applied to predict the occurrence, development, and prognosis of disease (Saberi-Karimian et al., 2021). In this study, based on 15 conventional indicators that have been reported to be related to aGVHD, machine learning algorithms were used to screen diagnostic biomarkers and construct a nomogram model to predict the probability of disease occurrence. Moreover, biomarkers associated with disease severity were also screened and nomogram was developed. These findings will help guide clinical practice.

## Methods

### Subjects collection

This study was approved by the Ethics Committee of Jinan Military General Hospital, and all patients signed written informed consent at the time of treatment in our hospital. From January 2016 to June 2018, patients with malignant and non-malignant hematologic diseases who underwent allo-HSCT in the Jinan Military General Hospital were retrospectively enrolled. Clinical diagnosis and grading of aGVHD were conducted according to the diagnostic criteria of aGVHD combined with histological verification (Rowlings et al., 1997). Meanwhile, according to the maximum grade of aGVHD, patients were divided into Grade I/II and Grade III/IV groups. Patients who received the T-cell-depleted graft/cord blood transplantation, developed primary graft failure, or had a history of graft rejection were excluded. Finally, 120 patients were enrolled, including 44 non-aGVHD and 76 aGVHD. In addition, 76 aGVHD were classified into 56 low (I/II) and 20 high (III/IV) grades.

### Allogeneic hematopoietic cell transplantation (allo-HSCT)

All the patients received bone marrow or peripheral blood stem cell transplantation. Before transplant, conditioning regimens were administered to patients, including high dose cyclophosphamide with busulfan or low dose busulfan with total body irradiation (TBI) 400 cGy. For GVHD prevention, recipients were given calcineurin inhibitor combined with methotrexate. In addition, anti-microbial drugs and ursodiol were used for preventing infection and cholestasis.

### Blood sample and clinical data collection

Whole blood samples were collected 2 days before transplantation, day 0, +14, +60 after transplantation. Blood samples were collected weekly after the diagnosis of aGVHD. In this research, blood samples within 24 h after transplantation were used for further analysis.

Standard biochemical indicators were measured by kits according to the manufacturers’ protocols. MILLIPLEX MAP Human cytokine/chemokine magenetic bead panel (Cat. No. HCYTOMAG-60K9) was used for measuring the concentrations of FMS-like tyrosine kinase-3 (Flt_3), IL-6, IL-7, IL-8, IL-9, IL-17A, interferon-gamma-inducible protein 10 (IP-10). lymphocyte activation gene 3 (LAG-3), toll like receptor 2 (TLR-2), programmed cell death protein 1 (PD-1), programmed death ligand 1 (PD-L1) were assayed by human immuno-oncology checkpoint protein magnetic bead panel (Cat. No. HCKPMAG-11K, Millpore). The concentrations of elafin, REG-3α, ST2, and TIM3 were tested by Elisa assay kit (Peviva AB, Stockholm, Sweden). The receiver operating characteristic (ROC) was used to evaluate the diagnostic performance of each index for aGVHD and non-aGVHD.

### Clinical characteristics screening and machine learning-based models

According to the different state of the patients, two prediction models related to disease occurrence (GVHD or non-GVHD) as well as disease severity (GVHD I-II or GVHD III-IV) were developed. Using the 15 clinical indicators collected above, two machine learning methods including Least Absolute Shrinkage and Selection Operator (LASSO) and random forest (RF) model were applied to select important predictors. As for the LASSO regression, the parameters of algorithm were optimized by 10-fold cross-validation using the R software glmnet package (version 4.1-6), and the λ value corresponding to the minimum error rate was calculated. For the RF model, the RF method in the R package random Forest (version 4.7-1.1) was used to filter the feature indicators, and then the obtained factors were sorted according to “Mean Decrease Accuracy” and “Mean Decrease Gini” (Han et al., 2016; Bénard et al., 2022). In this study, we opted “Mean Decrease Gini” method for the top 10 variables selection as previously described (Chen et al., 2022). Next, the characteristic indicators were obtained through intersection of the results selected by LASSO and RF algorithms. Next, based on the LASSO regression method, the risk score of each patient was calculated based on the expression level of characteristic indicators weighted by their coefficients (He et al., 2023; Yu et al., 2024). The formula is listed as follows (Wang et al., 2021; Shen et al., 2023):

Risk score=∑βgene×Expgene

β gene indicates gene regression coefficient and Expgene indicates gene expression level in each sample.

### Development and validation of nomogram model

As a convenient prediction tool, nomogram has become increasingly popular in medical research. It can assign a value to each influencing factor and then sum the scores to obtain a total value, thereby calculating the probability of clinical events (Park, 2018). In this study, nomograms were established using the rms package (version 3.5.0) based on the obtained predictors. To further evaluate the clinical application value of the model, the calibration curve was plotted using the calibrate function in the rms package.

### External validation

To further evaluate the predictive capability of the risk score model to classify aGVHD and non-aGVHD patients, aGVHD I/II and aGVHD III/IV patients, an external validation in an external cohort was conducted.

An independent cohort of another 10 non-aGVHD patients, 20 aGVHD patients (10 aGVHD I/II and 10 aGVHD III/IV) in the Shandong Cancer Hospital and Institute from January 2024 were recruited. The blood samples were collected in the same standard condition and the concentration levels of LAG-3, TLR-2, PD-1, Flt_3, IL-9, elafin, REG-3a and TIM3 were measured as described above. The risk score model was constructed and the diagnostic performance was evaluated by ROC curve analysis.

### Statistical analysis

All statistical analyses were conducted using the R package and GraphPad software. Results were expressed as mean ± standard deviation, and t test was used for comparison between two groups. For all analyses, *P* < 0.05 was considered statistically significant.

## Results

### Baseline and biochemical characteristics of patients

**FIGURE 1:**
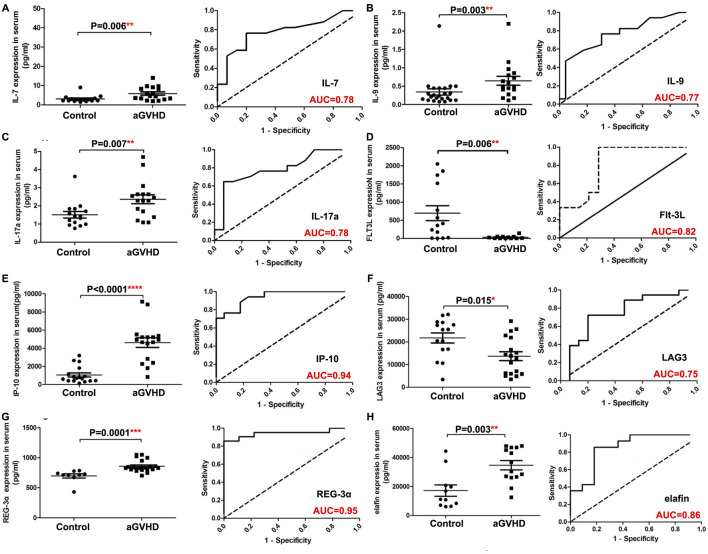
Comparison of serum indexes between non-aGVHD and aGVHD groups and evaluation of their diagnostic performance by ROC curve. **(A)** IL-7. **(B)** IL-9. **(C)** IL-17a. **(D)** Flt-3L. **(E)** IP-10. **(F)** LAG3. **(G)** REG-3α. **(H)** Elafin. T test was used for comparison between two groups.

**FIGURE 2:**
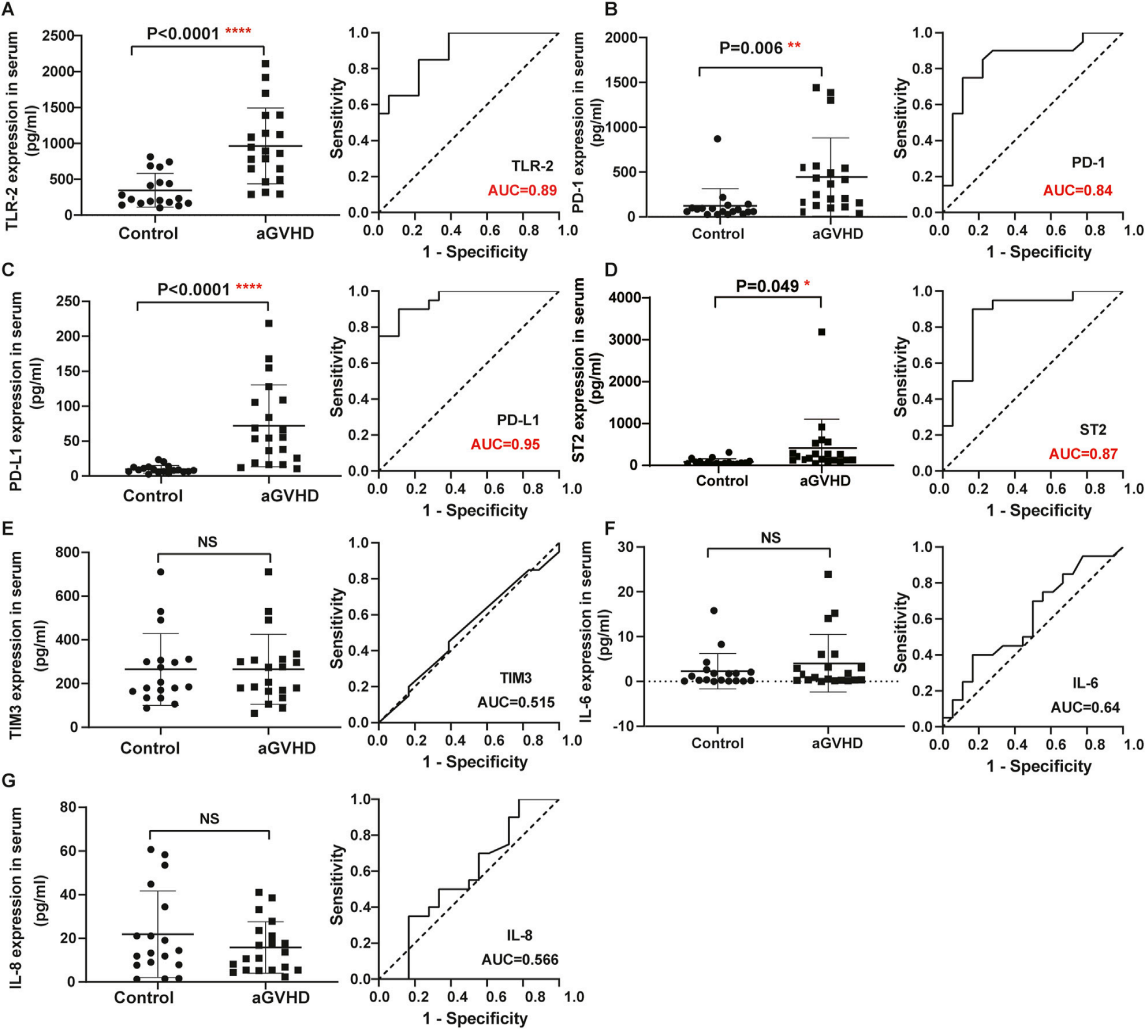
Comparison of serum indexes between non-aGVHD and aGVHD groups and evaluation of their diagnostic performance by ROC curve. **(A)** TLR-2. **(B)** PD-1. **(C)** PD-L1. **(D)** ST2. **(E)** TIM3. **(F)** IL-6. **(G)** IL-8. T test was used for comparison between two groups.

Detailed information on the baseline data of patients is listed in Table 1. Of the enrolled 120 allo-HSCT patients, 76 developed aGVHD, containing 56 patients with I/II grade and 20 patients with III/IV grade. The median age of aGVHD patients was 28 years (range 11–59 years). Compared with non-aGVHD patients, more aGVHD patients were in high risk (50 vs. 32) and malignant (65 vs. 35) status at the time of transplantation. Compared to the non-aGVHD group, the levels of IL-7, IL-9, IL-17a, Flt-3, IP-10, LAG3, REG-3α, TLR-2, PD-1, PD-L1, and ST2 were significantly elevated in the aGVHD group (*P* < 0.05, Figures 1, 2). ROC analysis showed that the AUC values of these indicators were all greater than 0.75, indicating excellent diagnostic performance (Figures 1, 2). However, no significant differences were observed in TIM3, IL-6, and IL-8 between two groups (Figures 2E–G).

**TABLE 1:** Clinical characteristics of enrolled patients.

Characteristics	Non-GVHD (n = 44)	aGVHD (n = 76)
Age, y		
Median (range)	27 (13–52)	28 (11–59)
Disease, n		
Malignant	35	65
Others	9	11
Disease status at allo-HSCT		
Low/mediate risk	12	26
High risk	32	50
Donor type		
Related	23	45
Unrelated	21	31
Regimen type		
Non-myeloablative	10	14
Myeloablative	34	62
Maximum aGVHD grade		
I-II	—	56
≥III	—	20
Organ target at aGVHD onset		
Skin	—	19
Gut	—	13
Liver	—	17
Multiple organs	—	27
aGVHD diagnosis day after HCT		
<15 days	—	10
15–42 days	—	33
>42 days	—	33

### Clinical screening model for aGVHD prediction

**FIGURE 3:**
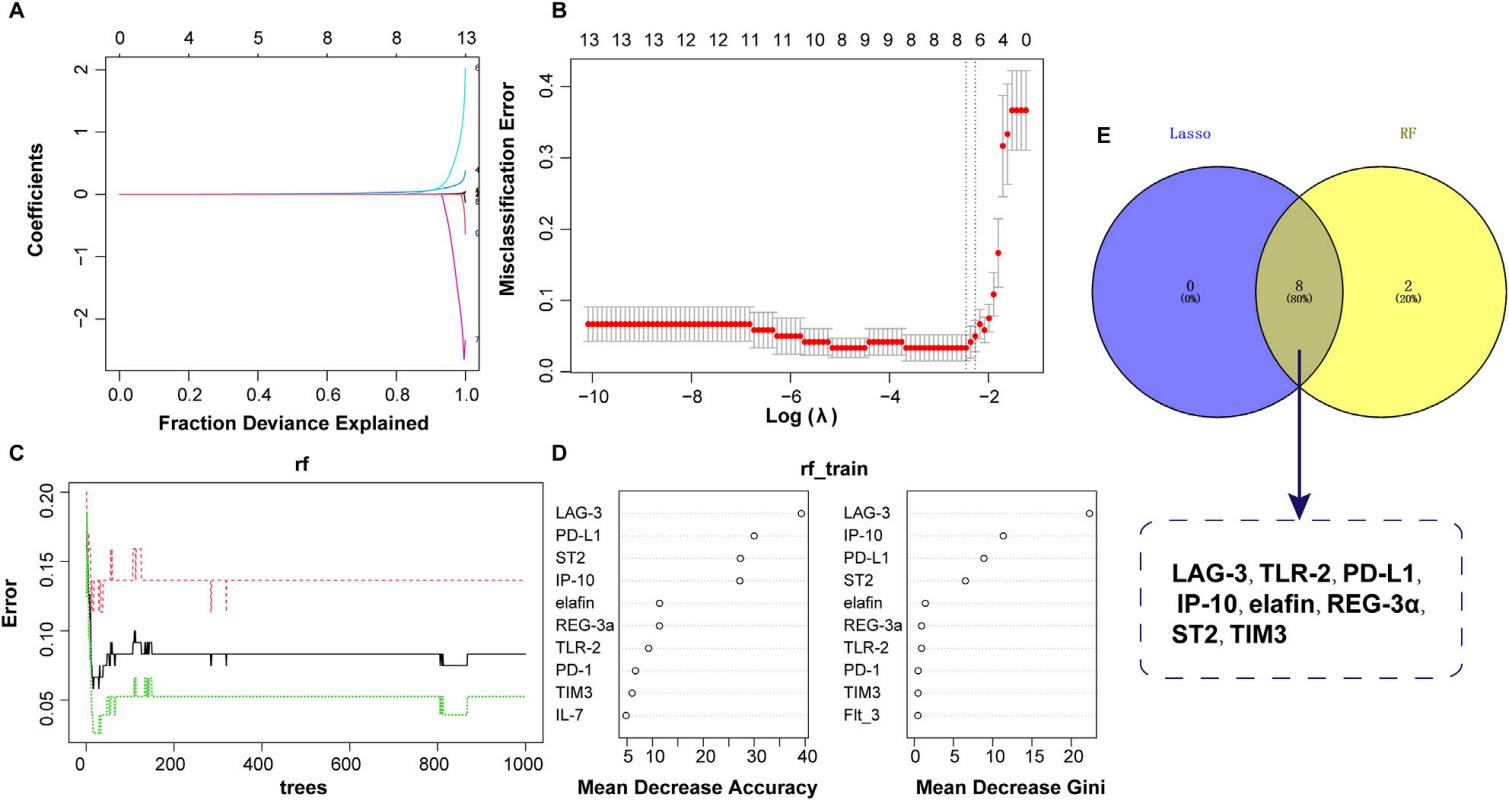
Predictor selection between non-aGVHD and aGVHD using LASSO and RF models. **(A)** Lasso coefficient profiles of the 8 clinical features. **(B)** Screening of optimal penalization coefficient lambda value in the LASSO model. **(C)** Correlation between error and tree number in the RF model. **(D)** Importance ranking of clinical features. **(E)** Venn plot revealing the intersection of biomarkers selected by LASSO and RF.

Based on the values of 15 clinical indicators from 120 samples, the LASSO regression prediction model was constructed to screen the characteristic variables. As shown in Figures 3A, B, eight significant variables were finally determined to be included in the LASSO model, containing LAG-3, TLR-2, PD-L1, IP-10, elafin, REG-3α, ST2, and TIM3. In the RF model, ten important factors in the RF regression were selected according to the Mean Decrease Gini rank, including LAG-3, IP-10, PD-L1, ST2, elafin, REG-3a, TLR-2, PD-1, TIM3, and Flt_3 (Figures 3C, D). Next, Venn diagram displayed the intersection of variables screened using LASSO and RF methods, and finally eight variables were obtained: LAG-3, TLR-2, PD-L1, IP-10, elafin, REG-3α, ST2, and TIM3 (Figure 3E).

### Diagnostic model based on nomogram

**FIGURE 4:**
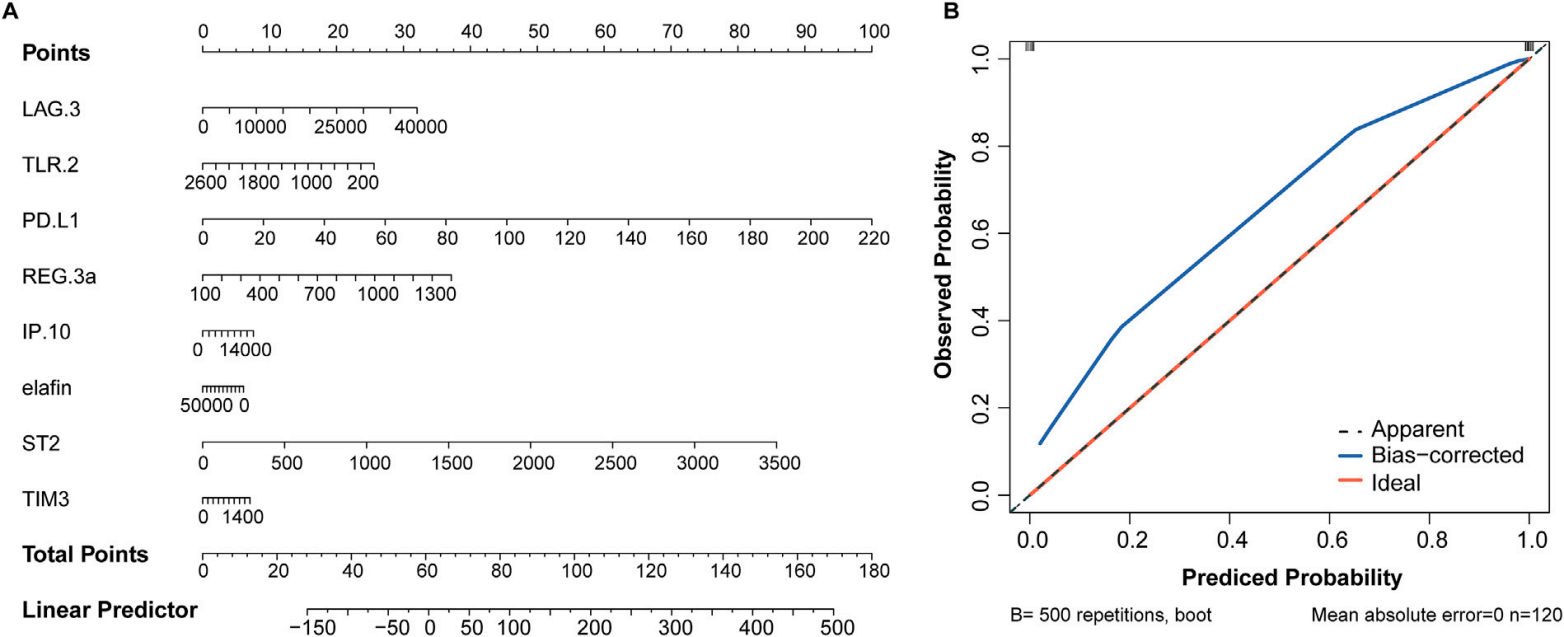
Nomogram based on 8 serum biomarkers to predict the occurrence of aGVHD. **(A)** Nomogram for predicting aGVHD probability. **(B)** Calibration curve for evaluating the predictive accuracy.

The eight variables selected above were included in the nomogram model, with PD-L1 and ST2 contributing more to event occurrence (Figure 4A). Calibration curve showed that the nomogram prediction results were in general agreement with the actual occurrence (Figure 4B).

### Clinical screening model for severity of GVHD (low and high grades)

**FIGURE 5:**
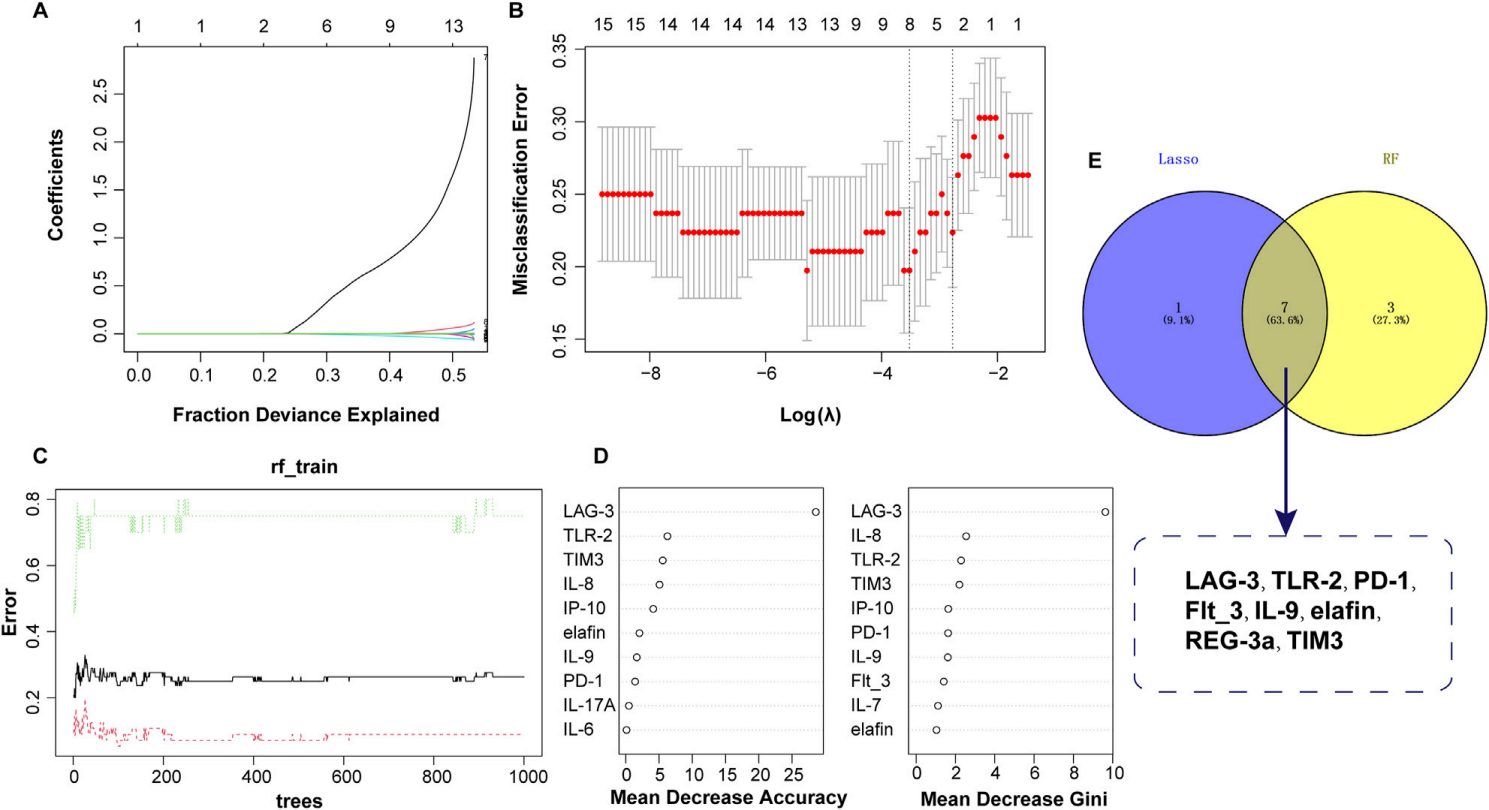
Identification of serum biomarkers related to aGVHD severity. **(A)** Lasso coefficient profiles of the 8 clinical features. **(B)** Optimal parameters selected by the LASSO model. **(C)** Relationship between error and tree number in the RF model. **(D)** Variable importance ranking in RF model. **(E)** Intersection of variables screened using RF and LASSO.

In order to further distinguish early and advanced GVHD diseases, two machine learning algorithms (LASSO and RF) were also used to screen diagnostic features. A total of 56 and 20 GVHD I-II and GVHD III-IV samples were included for analysis, respectively. As shown in Figures 5A, B, LASSO regression screened out the independent predictors of disease severity, and 8 of the 15 potential predictors were recommended: LAG-3, TLR-2, PD-1, Flt_3, IL-9, elafin, REG 3a, and TIM3. For the RF analysis, the top 10 most significant characteristic variables in the RF model were LAG-3, IL-8, TLR-2, TIM3, IP-10, PD-L1, IL-9, Flt_3, IL-7, and elafin, in order of Mean Decrease Gini (Figure 5C). Finally, seven optimal predictors of disease severity were selected by combining the results of the two detailed analyses above. These factors included LAG-3, TLR-2, PD-1, Flt_3, IL-9, elafin, and TIM3 (Figures 5D, E).

### Development of the predictive nomogram

**FIGURE 6:**
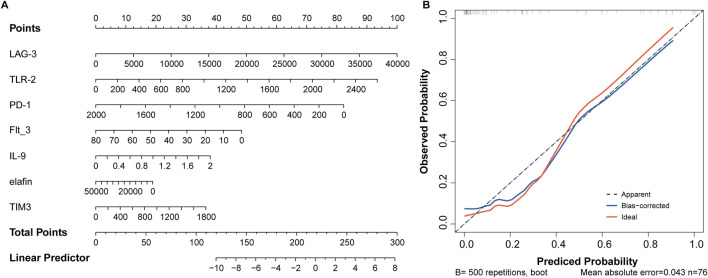
Seven factors establish a nomogram for aGVHD severity. **(A)** Nomogram model. **(B)** Assessment of predictive accuracy using calibration curve.

Based on the seven optimal predictors, a nomogram was constructed. As shown in Figure 6A, LAG-2, TLR-2, and PD-1 contributed higher values to the results. Meanwhile, the calibration curve showed that the bias-corrected line almost overlapped with the ideal line, indicating a satisfactory agreement between the predicted and observed results of disease severity (Figure 6B).

### Internal and external validation

**FIGURE 7:**
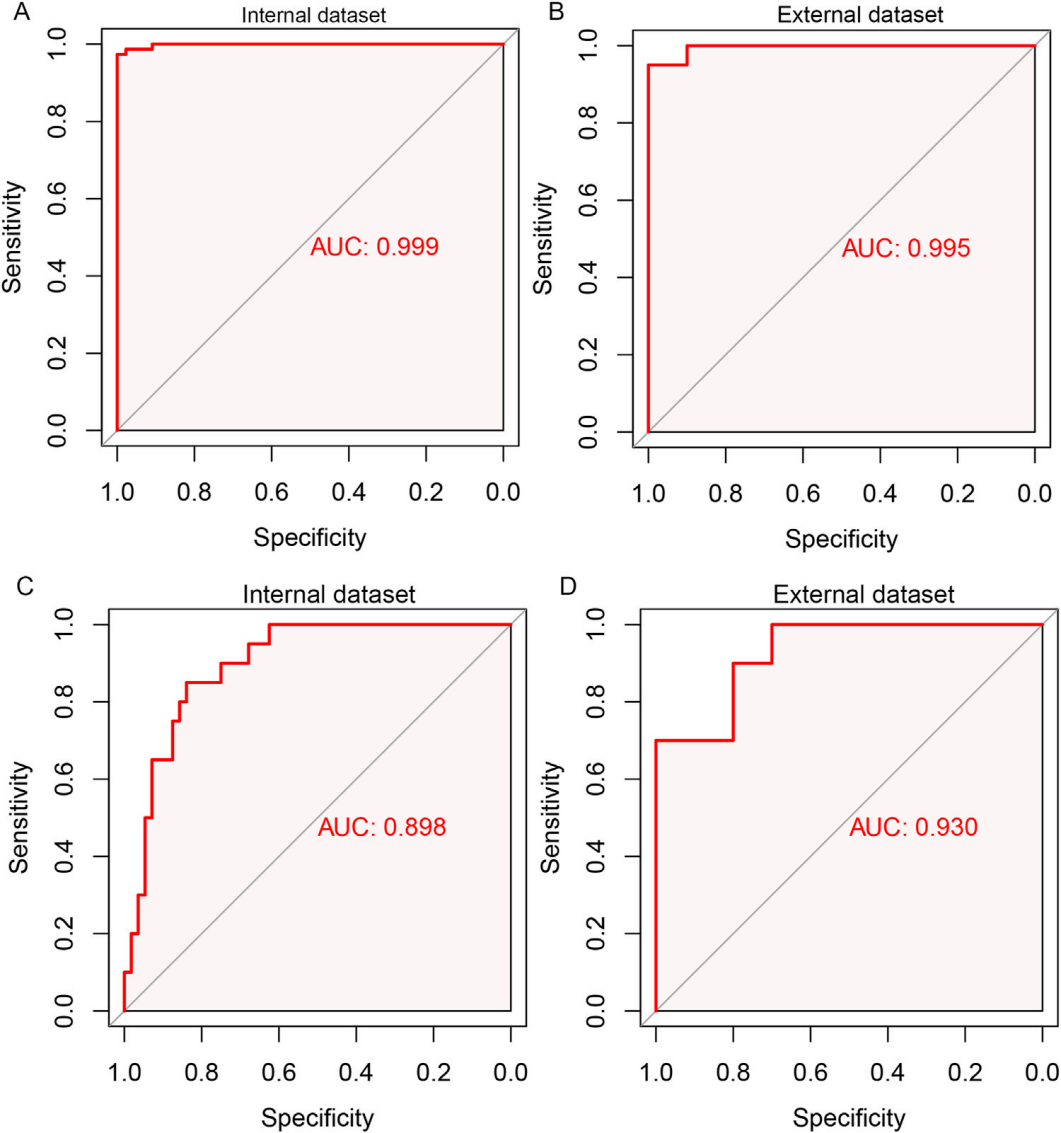
ROC curve analysis for the biomarker-based model for predicting aGVHD and its severity. Eight biomarkers-based diagnostic model for aGVHD in internal dataset **(A)** and external dataset **(B)**. Seven biomarker-based model for the severity of aGVHD in internal dataset **(C)** and external dataset **(D)**.

To further determine the validity of the candidate biomarkers, the biomarker-based risk score model was constructed and the predictive performance of the models was evaluated by ROC curve analysis. To distinguish aGVHD and non-aGVHD patients, eight biomarkers-based model was constructed. The model obtained an AUC of 0.999 in internal dataset and 0.995 in external dataset (Figures 7A, B). The seven biomarker-based model for classifying the severity of aGVHD was constructed. ROC analysis showed that AUC of the diagnostic model was 0.898 in internal dataset and 0.930 in external dataset (Figures 7C, D). These suggested that the biomarker markers obtained by machine learning methods exerted promising diagnostic value for aGVHD and its severity.

## Discussion

GVHD is a serious complication after hematopoietic cell transplantation, accompanied by increased morbidity and mortality (DiMaggio, 2020). Currently, the clinical benefits of GVHD treatment or prevention are limited (Holtan and MacMillan, 2016), and there is still a need to develop new methods for assessment and prediction of aGVHD. Clinical applications of biomarkers have been reported to be incorporated in the early stages after transplantation to aid detection and prediction of long-term clinical outcomes; biomarkers are also recognized as measures to predict the severity of aGVHD (Presland, 2017). However, single biomarker is limited for the sensitivity and specificity for the diagnosis or predictive value for aGVHD. A combination of several biomarkers for the diagnosis of aGVHD may be pressing.

The machine learning algorithms (such as LASSO and RF) have attracted widespread attentions in biomarker screening for the diagnosis and prediction of diseases (Wu et al., 2023). Cocho et al. have constructed the logistic regression model and identified the predictive biomarkers for ocular chronic GVHD (Cocho et al., 2016). LASSO regression and RF methods affords good predictive accuracy by running ten-fold cross validation, which may enhance the clinical diagnosis. The use of machine learning algorithms in identifying the serum biomarkers for aGVHD is rare. Thus, we developed models containing different serum indices through machine learning algorithms (LASSO and RF) to predict the biomarkers for the diagnosis and severity of aGVHD.

Our analysis revealed that LAG-3, TLR-2, PD-L1, IP-10, elafin, REG-3α, ST2, and TIM3 were diagnostic biomarkers for aGVHD. Part of the antitumor effect associated with allo-HSCT results from immune-mediated effects of donor T cells, which is commonly referred to as graft-versus-tumor (GVT) (Marmont, 1993). As attractive targets for modulating immune response, immune checkpoints (ICs) have been implicated in GVHD (Nguyen et al., 2020). Recent review has indicated that analogues of ICs exhibit anti-GVHD effects, and targeted drugs of ICs are a promising approach to balance the risk of GVHD with the effects of GVT (Zhu and Chen, 2023). As expected, we also found that several ICs, including PD-L1, LAG-3 and TIM-3, had diagnostic value for aGVHD. PD-L1 has been shown to selectively enhance T cell-mediated immune responses (Tumeh et al., 2014). Saha et al. revealed that in mice receiving PD-L1 −/− donor cells, reduction in inflammatory cytokine production, increase in apoptosis, and decline in aGVHD mortality without affecting graft-versus-leukemia response were observed, suggesting that interference with the PD-L1 pathway could serve as a potential therapeutic strategy to control or improve aGVHD (Saha et al., 2016; Schuchmann et al., 2008). The existence of homozygous T allele (rs870849) in LAG-3 impairs the inhibitory potential of the LAG-3 molecule in the allogeneic transplantation environment, leading to the increase of T cell response, which elevates the risk of severe aGVHD and reduces the survival rate of transplant patients (Cruz et al., 2023). TIM-3 is involved in the regulation of effector Th1 response, and its level was detected to be dramatically upregulated in liver CD8(+) T cells of aGVHD mice, indicating that it is implicated in the immune regulation of this disease (Oikawa et al., 2006). Increased TLR-2 contributes to rapid implantation and immune reconstitution after transplantation in mice, and it is a valuable target for improving transplantation efficiency without exacerbating aGVHD (Lee et al., 2015). IP-10 (also known as CXCL10) plays a central role in the pathogenesis of skin aGVHD by recruiting its ligand CXCR3(+) T cells to the sites of inflammation (Piper et al., 2007). Elafin, an epithelial protein secreted by keratinocytes in response to IL-1 and TNF-α, is overexpressed only in inflamed epidermis (Tanaka et al., 2000). It has been demonstrated to be an effective plasma biomarker for cutaneous aGVHD (Paczesny et al., 2010). REG3α is a biomarker of GVHD in the lower gastrointestinal tract, and plasma REG3α concentration also can predict the response to GVHD treatment and non-relapse mortality (Ferrara et al., 2011). Besides, soluble ST2 drives the transformation of helper T cells from type 2 to type 1 and is remarkably overexpressed in patients with aGVHD, indicating that it can be served as a predictive biomarker for aGVHD (Aladağ Karakulak et al., 2021). Taken together, these above evidences suggested that these factors may be directly involved in the pathogenesis of aGVHD.

Importantly, we also found that LAG-3, TLR-2, PD-1, Flt_3, IL-9, elafin, and TIM3 could be utilized as molecular markers of aGVHD severity. Among these, IL-9 and elafin have been confirmed to be significantly elevated in patients with grade III/IV aGVHD, which can be employed in the diagnosis and grading of aGVHD (Ito et al., 2020; Li et al., 2019). PD-1 expression in CD4(+) T cells was markedly correlated with the severity of aGVHD (Gallez-Hawkins et al., 2009). Besides, the upregulation of TIM3 on T cells is related to the severity of intestinal aGVHD, and extracorporeal photopheresis therapy can restore the immune system by reducing cytokines such as TIM3 and ultimately improve inflammation (Ni et al., 2022). These studies further confirmed that the markers we selected were indeed associated with the severity of aGVHD.

In this study, serum data from 120 patients were used to select the optimal aGVHD risk model. Our work has several strengths. First, few studies have evaluated the occurrence and severity of aGVHD using common hematological indicators. Second, two machine learning algorithms are applied to analyze the data, increasing the accuracy of the results. Finally, the constructed nomogram, especially the model for disease severity, can exhibit excellent predictive ability, which can provide a reference for clinicians in the diagnosis of aGVHD.

This work still has some limitations. First, retrospective studies may have led to subjective and selection biases that cannot be ignored. Second, all data came from a single hospital, which limited the predictors that could be included in the study. Finally, the clinical sample was limited and external validation of the model was lacking. Therefore, large-scale population samples need to be enrolled in the future to evaluate the diagnostic value of the constructed model for aGVHD patients.

## Conclusion

In short, the present findings suggest that the nomogram based on serum indicators for predicting the risk and severity of aGVHD has good calibration. Specially, seven key predictors were of great significant for diagnosis, including LAG-3, TLR-2, elafin, and TIM3. This model is expected to provide support for clinical practice and future research.

## Data Availability

The original contributions presented in the study are included in the article/supplementary material, further inquiries can be directed to the corresponding author.
